# The Value of Pretherapeutic Basal Calcitonin Cut-Offs for the Therapeutic Strategy and Prediction of Long-Term Outcome of Patients with Medullary Thyroid Cancer—A 30-Year Single-Center Experience

**DOI:** 10.3390/cancers16193343

**Published:** 2024-09-29

**Authors:** Martin B. Niederle, Teresa Binter, Philipp Riss, Bruno Niederle, Christian Scheuba

**Affiliations:** 1 Division of Visceral Surgery, Department of General Surgery, Medical University of Vienna, Waehringer Guertel 18-20, A-1090 Vienna, Austria; teresa.binter@meduniwien.ac.at (T.B.); philipp.riss@meduniwien.ac.at (P.R.); bruno.niederle@meduniwien.ac.at (B.N.); christian.scheuba@meduniwien.ac.at (C.S.); 2 Department of General Anesthesia, General Intensive Care and Pain Management, Medical University of Vienna, Waehringer Guertel 18-20, A-1090 Vienna, Austria

**Keywords:** medullary thyroid carcinoma, pretherapeutic calcitonin cut-offs, oncologic risk stratification, prognosis

## Abstract

**Simple Summary:**

Routine measurement of basal calcitonin (bCt) levels is used in the preoperative workup of thyroid nodules (“Ct screening”) and has been documented to facilitate the early diagnosis and treatment of patients with medullary thyroid cancer (MTC). Although clear cut-offs have been proposed, the relevance for predicting lymph node metastasis (LNM) and long-term outcomes (LOs) has so far not been tested on a large cohort of patients. In this study, 306 patients with MTC were grouped into three oncologic risk groups by clearly defined gender- and assay-specific bCt cutoffs. The rate of central LNM was 2.6% in risk Group 1 (minimal oncologic risk; recently published MTC incidence: females: 17.1%; males: 37.5%) and 6.0% in Group 2 (low oncologic risk: recently published MTC incidence 100%). Lateral LNM and distant metastasis (DMet) were not found. The overall cure rate for both groups was 95.7% and 20-year disease-specific survival (DSS) was 100%. In risk Group 3 (high oncologic risk) LNMs were found in 51.0% (thereof 88.9% also in the lateral neck compartment) and DMet in 13.5%. The cure rate dropped to 58.3% and DSS to 85.3%. Within a Ct screening program, grouping patients upon pretherapeutic bCt provides a simple risk classification system for indicating surgery and its extent, predicting LNM, and estimating LOs.

**Abstract:**

Background: The clinical relevance of clearly defined pretherapeutic basal calcitonin (bCt) cut-offs for predicting lymph node metastases (LNMs) and long-term outcomes (LOs) has so far not been tested in a large cohort of patients with medullary thyroid cancer included in a Ct screening program during the initial diagnostic workup of thyroid nodules. Material and Methods: Female (f) patients with a bCt level of ≤23 pg/mL and male (m) patients with a level of ≤43 pg/mL were assigned to Group 1 (minimal oncologic risk), patients with a bCt between 24 and 84 pg/mL (f) and 44–99 pg/mL (m) to Group 2 (low oncologic risk), and those with a bCt of ≥85 pg/mL (f) and ≥100 pg/mL (m) to Group 3 (high oncologic risk). All patients underwent surgery applying a uniform surgical protocol. The median follow-up was 100 months. Results: The study included 306 patients. In 3/115 (2.6%) patients in Group 1 and in 3/50 (6.0%) in Group 2, LNM in the central but not lateral neck and no distant metastases (DMet) were documented. In both groups, the biochemical long-term cure rate was 95.7% and the disease-specific-survival (DSS) rate was 100% at 10, 15 and 20 years. Lateral LNM and DMet were diagnosed only in Group 3. The bCt levels of N0 and N1 patients showed broadly overlapping ranges, thus impeding the differentiation between those patients through bCt. Both the cure rate and DSS were significantly worse in Group 3. The overall biochemical long-term cure rate was 78.2%. Conclusions: Within a Ct screening program, grouping patients upon pretherapeutic bCt provides a simple risk classification system for indicating surgery, predicting LN involvement, and LOs.

## 1. Introduction

Calcitonin (Ct) is the most important diagnostic biomarker for medullary thyroid cancer (MTC). Elevated basal Ct levels (bCt) identified during the routine biochemical workup of thyroid nodules (“Ct screening”) may be the first biochemical indicators of MTC. However, after excluding confounding factors [1], MTC is not responsible for a higher bCt in all patients. More often, a physiological increase in C-cells (C-cell hyperplasia [CCH]) may be verified after immunohistochemical examination of the thyroid gland [2]. Surgical overtreatment with possible side effects may be serious arguments against Ct screening. However, a definite cure of MTC can only be achieved by early diagnosis and early-stage adequate surgery before lymph node metastases (LNMs) or distant metastases (DMet) emerge [3,4].

In a previous study, we demonstrated that predefined gender-specific bCt cut-off levels are helpful in the early detection of MTC without stimulation tests [5]. MTC was documented histologically in all female and male patients (100%) with an assay-specific bCt > 23 pg/mL and >43 pg/mL, respectively. By contrast, in the group below these cut-off levels (the so-called “gray zone”), MTC (≤10 mm) was identified in 37.5% of the male and 17.1% of the female patients. Except for one hereditary female patient, no patients with LNM were documented within the gray zone [5]. Therefore, close surveillance with periodical bCt measurements seems justified to avoid overtreatment. Surgery is recommended when a significant increase in bCt is documented as exceeding the gender-specific cut-off levels [6]. In female and male patients with bCt > 85 pg/mL and >100 pg/mL, respectively, the number of those with LNM in the lateral neck increased significantly. LNMs were documented in at least 40% female and 52.9% male patients [5].

Including a larger cohort of patients, the aim of this comprehensive follow-up study was to demonstrate the relevance of using the proposed bCt cut-offs and clearly defining risk groups in the initial diagnosis of MTC in order to predict the extent of disease and long-term oncologic outcome.

Additionally, the applicability of the cut-off values for sporadic patients was to be tested and compared to hereditary (index) patients.

## 2. Patients and Methods

Since 1994, all eligible patients treated at the Medical University of Vienna have been included in a “Ct screening program” for the initial diagnostic workup of various thyroid nodules. All relevant patient documents with histologically verified MTC were collected prospectively.

Patients who exclusively had primary surgery and a complete set of diagnostic and clinical documents were included in the final analysis. Patients with reoperations were excluded [2].

All patients were tested during workup or postoperatively to evaluate their genetic backgrounds [7,8]. As genetic testing is time-consuming and costly, genetic results were not available in some patients before surgery, and therefore did not affect the therapeutic strategy. However, pheochromocytoma was excluded biochemically before surgery in all patients with suspected MTC. No patient included in this analysis was a member of a known MTC family at the time of surgery (no genetic screening patients included).

Following a standard operating procedure (SOP) [5], the diagnostic workup started with an ultrasound (US) of the neck to confirm one or more thyroid nodule(s) and to assess the status of cervical LN. However, the US findings did not influence further decision-making or the surgical strategy, as previous analyses had revealed unsatisfactory US sensitivity, particularly in detecting microMTC and (micro)LNM [9,10]. Also, neither fine-needle aspiration biopsy nor Ct measurements in wash-out fluid from fine-needle aspiration were performed during preoperative workup [11,12].

### 2.1. Calcitonin Measurements

Before 2006, the preferred assays for quantifying Ct were a two-site, immunoradiometric assay (Cis Bio International, Gifsur-Yvette, France) or a one-site, immunochemiluminometric assay (Nichols Institute Diagnostics, San Juan Capistrano, CA, USA). These two assays yielded the same cut-off levels (correlation coefficient 0.91) [13].

From 2006, an immunochemiluminescent assay (ICMA) produced by Diagnostic Products Corporation (DPC, Los Angeles, CA, USA), running as a fully automated test on a Siemens 2000 Immunoassay System (Siemens Health Care, Erlangen, Germany), was used for Ct analysis, as previously described in detail with a sensitivity of 2 pg/mL and reference values of up to 6 pg/mL for women and 8 pg/mL for men, respectively [14].

As proposed, a multiplication factor of 0.8 was applied to convert older assay results to DPC results in an attempt to facilitate comparability and overcome potential method-related bias [14].

Starting in 2014, Ct concentrations were determined with the Elecsys^®^ human CT assay (Roche Diagnostics, Basel, Switzerland), a one-step sandwich assay based on the streptavidin-biotin technology. Values from the Roche Cobas assay were subsequently converted to values of the DPC assay using the recommended appropriate conversion factor to enable a direct comparison in this study [15].

This assay system was applied as the ICMA by DPC is used worldwide and the results of the current investigation are more easily comparable to other published studies.

Patients were eligible for surgery if persistently elevated bCt levels, exceeding the reference values in at least two measurements, were documented after excluding those with marked reduced renal function (glomerular filtration rate <45 mL/min/1.73 m). As neither pentagastrin nor calcium stimulation tests were seen to improve the diagnosis, the indication for surgery was exclusively based on bCt [5,16]. Only bCt values were used for the present detailed analysis and to group the patients into risk Groups 1–3 (see below).

### 2.2. Surgery

(Total) thyroidectomy and (first-step) bilateral micro dissection of the central neck (CND) (level 6, C1a + b) were performed in all patients [17,18,19].

In 5 patients, hemithyroidectomy and unilateral CND were performed only due to solitary thyroid nodules and mildly elevated bCt. MicroMTC was found incidentally in the course of postoperative immunohistologic workup with subsequently immeasurable post-Ct [9]. These patients were also included in the analysis.

In accordance with the surgical part of the SOP, systematic (“functional”) microdissection of both lateral neck LN compartments (BLND; levels 2–5; C2, C3) [17,18] was performed in patients with clinically apparent (macro)tumors (pT1b-4).

Transcervical mediastinal dissection (level VII [17]) was performed in 2 patients with enlarged LNs in the upper thoracic outlet. Transsternal mediastinal dissection (C4) [18] was carried out exceptionally in 3 patients with huge mediastinal LNMs to avoid vascular complications.

In selected patients with documented DMet (M1) at the time of diagnosis, thyroidectomy was performed with bilateral CND and extirpation of macroscopically enlarged uni- or bilateral LNs only.

### 2.3. Histology

In keeping with the pathological part of the SOP [20], the en bloc removed thyroid gland and all LN specimens were subjected to pathological examinations. The surgeon provided the pathologist with the basal Ct levels before initiating histological examinations, thus serving to look more carefully for C-cell abnormalities (CCH, MTC). By definition [21], MTC was identified if a focal loss or reduplication of the basement membrane was observed upon immunohistochemistry.

Tumor grading (high grade, low grade), as recommended recently for predicting individual outcome [22], did not affect surgery as this information is usually not available intraoperatively.

The demographic data were documented prospectively and recorded in a specially compiled database, including gender and age, genetic findings, levels of preoperative bCt, and post-Ct at the time of the last follow-up, and histological findings, e.g., tumor size (in mm), number of affected and removed LNs in each compartment, and tumor stage based on the TNM classification of the American Joint Committee on Cancer staging system [23].

### 2.4. Follow-Up

Post-Ct levels were determined after 1, 6, and 12 months and annually thereafter. In patients with elevated post-Ct, US was performed to detect local structural persistence or recurrence. Functional imaging (F-DOPA-PET-CT) was applied in the case of post-Ct > 150 pg/mL.

Patients were considered to be definitively “cured” when post-Ct levels remained permanently undetectable (<2 pg/mL). Patients were referred to as “likely disease-free” when showing post-Ct levels that were detectable (>2 pg/mL), yet below the upper normal gender-corrected Ct limits without evidence of structural recurrence and without an increase in post-CT levels over time. Post-Ct levels higher than the gender-corrected upper normal bCt level with/without structural recurrence were classified as “persisting disease”. “Recurrence” referred to post-Ct levels <2 pg/mL during a follow-up of at least 12 months and afterwards a slight or rapid increase with/without structural recurrence.

### 2.5. Statistical Analysis

Statistical analyses were performed with IBM SPSS 28.0 for Windows and Microsoft Excel for Windows Version 2404.

As the continuous parameters were not normally distributed, the parameters are presented as the median and interquartile range (IQR; 25th–75th percentile) or absolute range (min–max). The Wilcoxon signed-rank test was performed for group comparisons of continuous parameters. Binominal parameters are presented as absolute numbers and percentages. To compare group differences for dichotomous proportions, Fisher’s exact test (2 × 2 or R × 2) was conducted due to an inadequate sample size for the chi-square test of homogeneity, as established by Cochran [24]. No test was carried out in the case of 2 × 2 crosstabulation with more than one group with zero group members.

Kaplan–Meier survival analyses [25] were conducted to present and compare disease-specific survival (DSS) within different subgroups of the collective. Patients at risk and DSS (percentage with 95% CI [1.96 × cumulative standard error]) are given for each survival analysis. Log-rank tests were conducted to determine whether there were differences between the groups. A two-sided *p*-value of ≥0.05 was considered as not significant.

## 3. Results

All 306 patients had histologically confirmed MTC. Two hundred and sixty-six patients (86.9%; m: 129 [48.5%]; f: 137 [51.5%]) had sporadic disease (Table 1). One of the various RET proto-oncogene mutations was documented postoperatively in 40 (13.1%) patients (hereditary “index” patients = first affected member of a family; m: 13 [32.5%]; f: 27 [67.5%]; see Supplemental Table S1).

MTC was classified [23] as pT1a in 196 (64.1%) of 306, as pT1b in 51 (16.7%), as pT2 in 43 (14.1%), as pT3 (a/b) in 11 (3.1%), and as pT4a in 5 (1.6%) patients. No pT4b tumors were documented.

There were no statistically significant group differences between patients with sporadic or hereditary MTC for the distribution of gender, median age, bCt, tumor diameter, T classification, or presence of metastases (*p* = n.s. for all). In all risk groups (see below), multiple tumors were documented significantly more often in hereditary patients (Table 1; Group 1: *p* = 0.024; Groups 2 + 3: *p* < 0.001).

At the time of diagnosis, DMet were documented in 19 (6.2%) of 306 patients (solitary or multiple: lung [n = 7], liver [n = 9], and/or bones [n = 5]).

The median bCt level was significantly higher in M1 patients (M1: 7114 [IQR: 1942–17,813; min–max: 115–35,091 pg/mL]; M0: 54 [IQR: 17–416; min–max: 1–22,945 pg/mL]; *p* < 0.001).

LNMs were found in 78 (25.5%) of the patient total (N1a: 14/306 [4.6%]; N1b: 64/306 [20.9%]).

Excluding patients with M1, the bCt levels of N1 patients were significantly higher than those of N0 patients (N0: 36 [IQR: 15–118] pg/mL; N1: 476 [IQR: 146–1188] pg/mL; *p* < 0.001).

Complete follow-up data were obtained for 303 patients (median follow-up: 100 [IQR: 53–159] months). Three patients were lost to long-term follow-up.

### 3.1. Comparison of Disease-Specific Survival in Sporadic and Hereditary, N0 vs. N1 and M0 vs. M1 Patients

The median follow-up time of sporadic patients was 93 (IQR: 44–151) months and that of hereditary (index) patients was 150 (IQR: 83–197) months.

**Figure 1:**
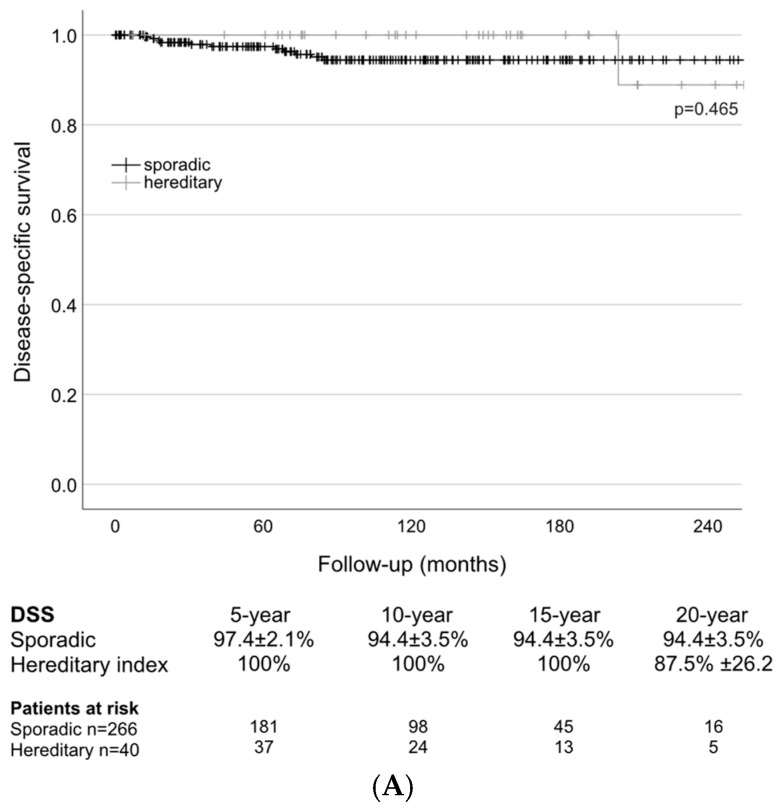

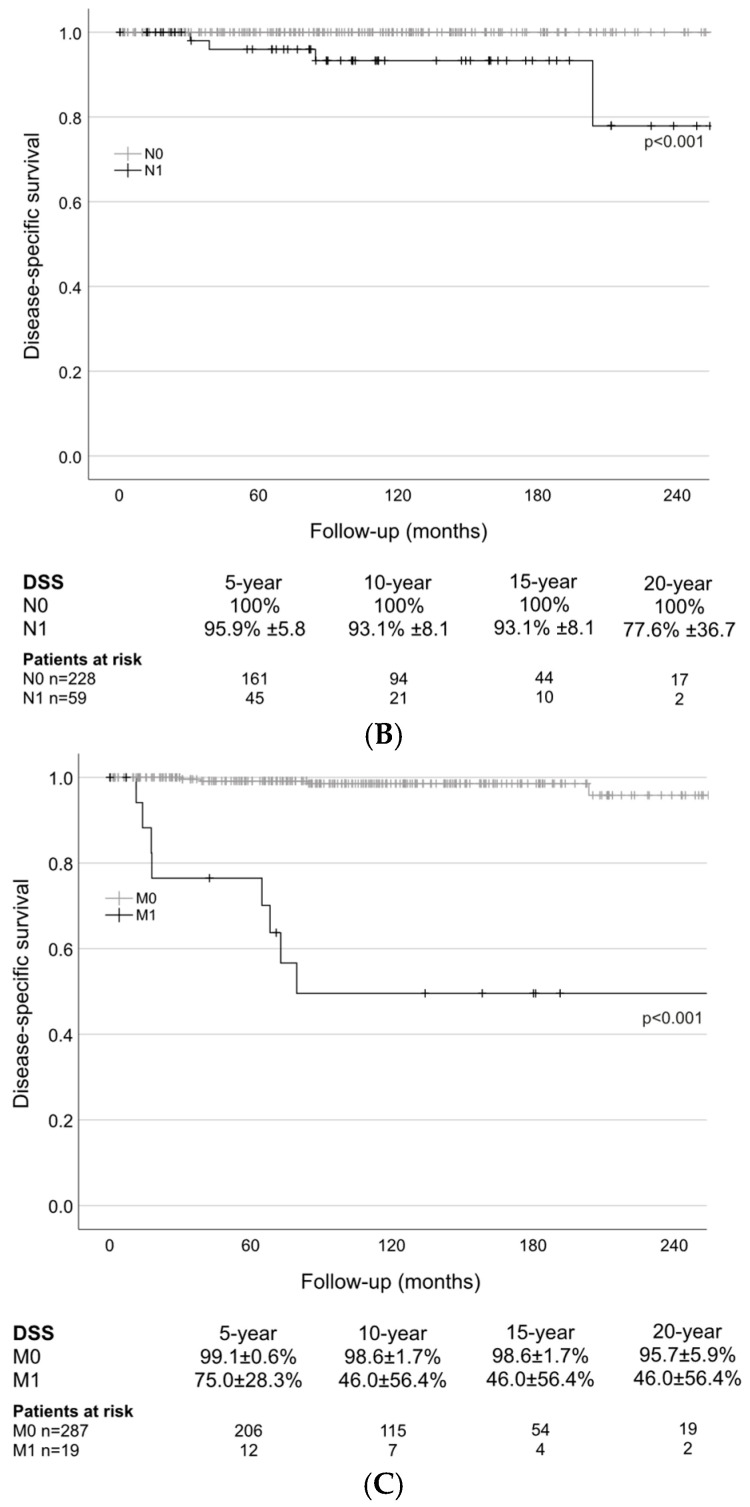
(**A**) Long-term disease-specific survival (DSS) of sporadic and hereditary (index) patients. (**B**) Long-term disease-specific survival (DSS) of patients with (N1) and without (N0) lymph node metastases (M0 only). (**C**) Long-term disease-specific survival (DSS) of patients with (M1) and without (M0) distant metastases.

The estimated DSS rate for sporadic patients at 5 years was 97.4% and then 94.4% at 10, 15, and 20 years and 100% at 5, 10, and 15 years, and 87.5% at 20 years for hereditary patients. There was no significant difference between the two variants (*p* = 0.465; Figure 1A).

The median follow-up time was 101 (IQR: 51–157) months for N0 and 100 (IQR: 66–159) months for N1. The estimated 5-, 10-, 15-, and 20-year DSS rates of N1 patients were 95.9%, 93.1%, 93.1%, and 77.6%, respectively, while the overall survival rate of N0 patients was 100% for all four periods (*p* < 0.001; Figure 1B).

The median follow-up time was 100 (IQR: 55–159) months for M0 and 71 (IQR: 18–180) months for M1. The estimated 5-, 10-, 15-, and 20-year DSS rates of M0 patients were 99.1%, 98.6%, 98.6%, and 95.7%, respectively. For patients with M1 upon initial diagnosis, the 5-year DSS rate was 74.0% and 46.0% in the three following periods (*p* < 0.001; Figure 1C).

### 3.2. Pretherapeutical bCt Cut-Offs Characterizing Oncologic Risk Groups

To predict the possible clinical outcome at initial diagnosis, the patients were assigned to one of the three oncologic risk groups defined by the gender-specific bCt levels, as developed in previous studies [5,16].

Patients with a bCt of ≤23 pg/mL (f) and ≤43 pg/mL (m) were assigned to Group 1 (minimal oncologic risk), those with a bCt of 24–84 pg/mL (f) and of 44–99 pg/mL (m) to Group 2 (low oncologic risk), and those with a bCt ≥ 85 pg/mL (f) and ≥ 100 pg/mL (m) to Group 3 (high oncologic risk).

The histological findings in the thyroid gland and the presence and location of LNM were documented for each patient in each group and the clinical outcome and long-term survival rates were compared between the groups.

Factors that possibly influence long-term survival, such as bCt, presence of LNM, and DMet, were distributed homogeneously between sporadic and hereditary patients within the three groups (Table 1). Therefore, identical bCt cut-offs were applied for sporadic and hereditary patients and both were analyzed as one in each of the three groups.

#### 3.2.1. Group 1—Minimal Oncologic Risk

One hundred and fifteen (37.6%) patients were included in Group 1 (gray zone, mildly elevated bCt). Except in one male patient (0.9%; bCt 22 pg/mL; pT1b [11 mm] pN0 [0/3)]), all other patients’ 114 (99.1%) tumors were histologically classified as pT1a (≤10 mm; Table 1).

While 112 (97.4%) patients were classified as pN0, central LNMs (pN1a) were verified in 3 (2.6%) patients. In two men (bCt: 12 and 19 pg/mL; both sporadic/multifocal) and one woman (bCt: 23 pg/mL, hereditary/unifocal), only one single LNM was documented unilaterally in the central neck (Supplemental Table S2).

No patient showed DMet at the time of diagnosis.

**Figure 2:**
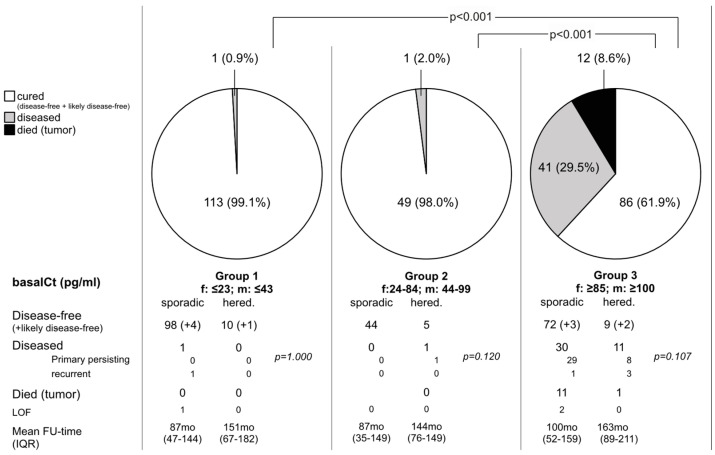
Long-term follow-up of patients assigned to three oncologic risk groups: Group 1—minimal oncologic risk; Group 2—low oncologic risk; Group 3—high oncologic risk; f: female; m: male; hered.: hereditary, LOF: loss of follow-up; FU: follow-up; Mo: month.

One patient was lost to follow-up. All the others were followed over a median of 94 (IQR: 51–148) months. By the last follow-up, 108 (94.7%) patients were biochemically cured (alive: n = 88; died of other reason: n = 20). Five patients (5/114; 4.4%) were clinically and radiologically free of disease, but bCt was measurable within the gender-specific normal levels (likely disease-free). One patient (0.9%; follow-up 177 months) showed “recurrent disease” with an asymptomatic clinical course. The patient had shown 1 LNM out of 166 dissected LNs. He was biochemically disease-free for 12 months (bCt: <2 pg/mL), subsequently showing a slow step-by-step increase up to 16 pg/mL within the following 143 months and remaining stable for the last 24 months (Figure 2).

#### 3.2.2. Group 2—Low Oncologic Risk

In total, 50 (16.3%) of 306 patients belonged to Group 2, where 47 (94%) tumors were classified as pT1a and 3 (6%) as pT1b.

In 3 (6.0%) of 50 patients (one male, two female) with pT1a tumors, histological workup verified one single central LNM metastasis per patient (Supplemental Table S2).

No patient showed DMet at the time of diagnosis.

During a median follow-up of 88 (IQR: 42–149) months, 49 (98.0%) of 50 patients were biochemically cured (alive: n = 37; died of other reason: n = 12). One patient showed persisting disease (follow-up time 76 months; non-tumor-related death; last post-CT 8 pg/mL without evidence of structural recurrence; Figure 2).

#### 3.2.3. Group 3—High Oncologic Risk

Group 3 consisted of 141/306 (46.1%) patients. The T classifications are summarized in Table 1.

Sixty-nine (48.9%) patients were classified as pN0, while 72 (51.1%) were documented as pN1 (pN1a: n = 8; pN1b: n = 64; central + unilateral: n = 34; central + bilateral: n = 15; central negative + unilateral positive = “skip” LNM: n = 15). Details concerning the pattern and frequency of LNM in relation to pT classification are presented in Supplemental Table S3. The proportion of patients with N1 was significantly higher in Group 3 than in Groups 1 and 2 (*p* < 0.001 for both).

The total number of resected LN ranged from 1 to 200 (min–max) with a metastatic LN ratio of 0–0.824 (min–max). A median of 60 (IQR: 13–98) LNs were dissected.

**Figure 3:**
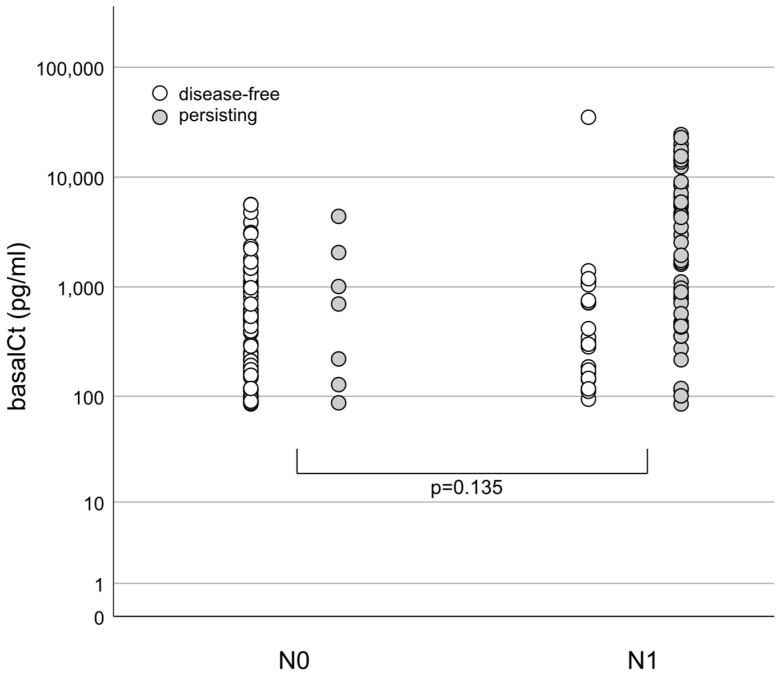
Group 3—oncologic high-risk; individual bCt levels (pg/mL)—presence of lymph node metastases and long-term outcome. M1 excluded; N0: no lymph node metastases where n = 69; N1: lymph node metastases where n = 53; bCt: N0: 511 (IQR: 156–1034) pg/mL, N1: 715 (IQR: 284–1400) pg/mL (*p* = 0.135).

Excluding M1, the bCt levels of patients with N0 and N1 showed no significant difference, but there was a broad overlap (Figure 3). It was impossible to preoperatively differentiate between patients with and without LNM.

The 19 (13.5%) patients with DMet were documented exclusively in Group 3 and all had LNM.

Details concerning the pattern and frequency of LNM and DMet in relation to pT are shown in Supplemental Table S3.

In total, 81 (58.3%) of 139 patients (lost to follow-up: n = 2) were cured (bCt < 2 pg/mL), while post-Ct was documented in the normal range (likely disease-free) in 5 (3.6%) patients and 41 (29.5%) patients had disease (persisting: n = 37; recurrent: n = 4; follow-up 104 [IQR: 65–166] months). Twelve patients died of conditions related to MTC (Figure 2).

### 3.3. Disease-Specific Survival and Outcome in the Three Risk Groups

**Figure 4:**
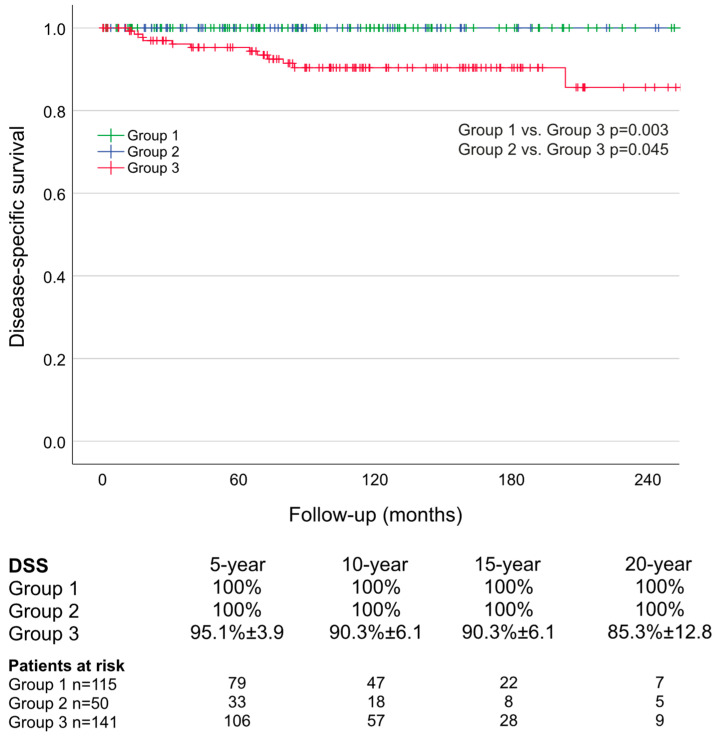
Long-term disease-specific survival (DSS) of patients assigned to oncologic risk Group 1, Group 2, and Group 3.

The estimated 5-, 10-, 15-, and 20-year DSS rates were 100% in Groups 1 and 2 and 95.1%, 90.3%, 90.3%, and 85.3%, respectively, in Group 3. DSS was significantly better in Groups 1 and 2 as compared to Group 3 (Group 1 vs. Group 3: *p* = 0.003; Group 2 vs. Group 3: *p* = 0.045; Figure 4).

The median follow-up of patients in Group 3 was 104 (IQR: 65–166; 5th–95th: 11–252; min–max: 1–325) months. The long-term cure rate of N0 patients was 88.4% (61/69 patients; likely disease-free: n = 1; persisting disease: n = 3; recurrent disease n = 4). In the four patients experiencing recurrence, increase in post-Ct was diagnosed 60, 60, 132, and 144 months after surgery (bCt at last follow-up: 8, 8, 7, and 7 pg/mL). No patient demonstrated local structural recurrence. The overall cure rate was significantly better in Groups 1 and 2 than in Group 3 (*p* < 0.001; Figure 2). In total, 21 (30.0%) of 70 patients with positive LN (N1a and N1b) were biochemically cured (persisting disease: n = 37; tumor-associated death: n = 12).

## 4. Discussion

“Ct screening” facilitates early diagnosis of MTC in patients with elevated bCt undergoing evaluation for thyroid nodules and an adequate, stage-adapted, less extended surgical approach, thus improving cure rates and long-term survival [3,4,26].

Suspecting MTC, all patients were treated according to the same surgical protocol following a guideline-adapted approach [27], which was modified by initial first-step bilateral CND without increasing local complications [19] and also the same histopathological workup of the specimens [20]_._

CND and BLND were performed because of the low sensitivity of US to detect LNM in the central and lateral neck (central neck: 6%; lateral neck: 56%; overall 43%) [10], in particular to verify micro-metastases, regardless of the results of all available diagnostic possibilities. This uniform surgical procedure yielded a risk classification and prediction of LO correlating pretherapeutic bCT levels, LN pattern and frequency.

As reported recently [28], the stage-dependent 5-, 10-, and 15-year cumulative disease-free survival rates were 61.8%, 48.6%, and 38.2%, respectively. In another study [29], DSS was reported after 5, 10, 15, and 20 years at 82%, 75%, 71%, and 69%, respectively.

The survival data and cure rates as an effect of Ct screening and early diagnosis of MTC in this cohort seem much better comparable to the literature. The DSS rates of patients with sporadic tumors were 94.4% after 10, 15, and 20 years with no significant difference compared to hereditary (index) patients.

“Cure” is not defined uniformly in the literature [30]. In the current analysis, only patients with permanent immeasurable post-Ct levels were considered “cured”. This is in contrast to most published reports defining patients as “cured”, when post-Ct levels were in the assay- and gender-specific normal ranges. Over a median follow-up of 100 months with regular clinical and biochemical check-ups and a strict definition of cure (post-Ct: not measurable), surgery served to cure 78.5% (238/303) in the long term.

Strict rules must be followed when interpreting pretherapeutic bCt to avoid misinterpretations. The normal upper limits are assay-dependent and vary across gender with female patients having slightly lower bCt levels than male patients [16]. Higher biomarker levels are pathognomonic for more advanced disease correlating with the extent of MTC [31,32], more directly with tumor size but less clearly with LN involvement [33].

Diagnostic uncertainty remains in patients with bCT levels “mildly elevated” above the assay- and gender-specific normal range, which may be caused by CCH or microMTC. Neither basal nor stimulated Ct levels in either gender make it possible to correctly discriminate between the two [2,9,16]. Taking all considerations into account, an appropriate bCt cut-off seems to be the major factor to positively affect diagnostic efficiency in correctly predicting MTC and the extent of LN involvement, and in assessing the patients’ oncologic risk, thus avoiding unnecessary and overextensive interventions.

In the current study, previously collected results [5] were used to retrospectively assign a large number of patients with MTC to three oncologic risk groups (minimal, low, high) on the basis of pretherapeutic gender-specific bCt in an attempt to improve prediction of individual oncologic risk at the time of diagnosis.

In patients with bCt levels between the upper limit of normal and slightly below the gender-specific cut-off level (gray zone; mildly elevated: ≤23 pg/mL [f], male ≤43 pg/mL [m]), MTC was identified in 17.1% of the women and in 37.5% of the men [5]. There are currently no clear recommendations for the optimal management of patients with “mildly elevated” bCt (gray zone; group 1) because a definite diagnosis of MTC is not possible. Machens and Dralle [33] concluded that if MTC is found in male and female patients with bCt < 20 pg/mL (applying the same Ct assay as in this study), MTC is always ≤10 mm and is limited to the thyroid gland without central LNM, thus making prophylactic CND unnecessary [33]. However, Parks et al. [34] recently reported central LNM in 2/22 (9.1%) patients with bCt levels of up to 20 pg/mL (applying a two-site immunoradiometric assay; Medgenix CT-U.S.-IRMA kit, BioSource Europe S.A., Nivelles, Belgium). In the current study, thyroidectomy and initial bilateral CND revealed unilateral central LNM in two male patients, both with sporadic and multifocal microMTC, and in one female patient with hereditary, unifocal MTC (overall 3/115; 2.6%) in Group 1 (minimal oncologic risk). All patients were cured with the exception of one (1/114; 0.9%) N1 patient showing late biochemical recurrence. Although clinical experience is limited in patients in the gray zone, [35], Frank-Raue et al. [6] and Broecker-Preuss et al. [36] proposed “active surveillance” as an acceptable alternative to surgical (over)treatment. They recommended that medical professionals should biochemically re-evaluate these patients in intervals of 3–6 months and advised surgery in the event of rising bCt, which may indicate MTC. However, if patients request surgery even after careful information about the “minimal oncologic risk” and the good LO of patients with mildly elevated bCT as well as the potential morbidity of surgery, total thyroidectomy with bilateral CND should be recommended in the light of the current analysis.

In a recently published review aimed to elucidate the prevalence and significance of “indeterminate Ct values” (10–100 pg/mL), the authors concluded that the rate of MTC cannot be overlooked with the risk of MTC increasing significantly when bCt is above 20 pg/mL. However, gender-specific differences or surgical details were not considered [37].

Under strictly standardized conditions (assay, age, gender), MTC can be predicted without false-positive results in either gender [5]. The patients included in Group 2 (low oncologic risk; bCt: 23–85 pg/mL [f], 44–99 pg/mL [m]) definitely had MTC, possibly central (non-lateral) LNM and no DMet. In 3/50 (6.0%) patients, LNMs in the central but not in the lateral neck were identified. All patients were cured with the exception of one. The patient with mildly elevated post-Ct (8 pg/mL) showed no structural persistence. Thus, applying this risk group stratification, these patients would have been candidates for thyroidectomy and for initial bilateral CND only, thus avoiding LND.

The findings are in contrast to those reported by Machens et al. as well as Park et al. who documented lateral LNM in patients with bCt levels < 100 pg/mL. Machens et al. [33] reported central and lateral LNM on the side of the MTC and even both central and contralateral LNM in 8/58 (13.8%) patients. Park et al. [34] reported central and ipsilateral LNM in 1/22 (4.5%) patients. However, in contrast to this analysis, neither group applied gender-specific cut-offs and Park et al. did not mention in detail whether Ct measurements were performed for initial or recurrent diagnosis.

Based on higher bCt levels (f: ≥85 pg/mL; m: ≥100 pg/mL [5]), the patients in Group 3 were correctly assessed as high-risk patients. All 19 (100%) patients with DMet and 72/78 (92.3%) patients with LNM (N1b: 64/72 [88.9%]) were categorized into this group.

Due to irregularly overlapping bCt levels, it was impossible to correctly discriminate patients with N0 from those with N1a and/or N1b. The patterns of LNM distribution (central with/without lateral LNM and the various combinations of distributions) varied between patients and were not related to size and tumor extension expressed by the pT classification. Unilateral and bilateral LNMs were observed even in patients with uni- or bilateral tumors and could not be predicted by bCt [38,39].

Besides tumor size, other tumor-associated factors, e.g., grading (differentiation, ability to produce Ct), morphological and biological differences [22,40], or occult, undiagnosed DMet, may affect individual bCt levels and may impede the correct prediction of LNM. This fact complicates planning of the extent of LN surgery. The early appearance, frequency and pattern of LNM do not correlate with the T classification of the primary tumor. Even if central LNs are negative in frozen sections, involved lateral LNMs (“skip LNM”) may occur and were identified in 15/141 (10.6%) patients. This phenomenon has been described in the literature in up to 35% [41,42].

An overall long-term cure rate of 58.3% was achieved in Group 3. Only one patient in Group 1 (primarily classified as pN1a) and four patients in Group 3 (primarily classified as pN0; together 5/303 [1.7%]) showed biochemically “mild recurrence” between 60 and 177 months after surgery. None showed evidence of structural disease. Late recurrence has been reported in the literature in 3.3% of the patients of any stage and even following perceived “complete” primary surgery [43].

One limitation of this retrospective study was that occult distant metastases were not excluded (inaccessibility of functional imaging) in all patients with higher pretherapeutic bCt levels. Additionally, primary-tumor grading [22] was not available in all patients, which potentially could have helped to better explain the correlation between bCt and the frequency/distribution of LNM and DMet in Group 3. However, tumor grading was not available intraoperatively because it is based on morphologic assessment in conjunction with Ki67 immunohistochemistry and thus cannot assist in individualizing the surgical approach pre- or intraoperatively.

## 5. Conclusions

To the best of our knowledge, this is one of the largest single-center, long-term follow-up studies of patients with MTC diagnosed within a “Ct screening program”. By simply taking venous blood samples for bCt measurements (using a standardized modern Ct assay- and gender-specific thresholds), patients with elevated bCt can be subdivided into pretherapeutic risk groups. On the one hand, early MTC diagnosis as well as precise predictions of the extent of disease and LO are facilitated. On the other hand, this approach could facilitate the application of a more individualized (tailored) therapeutic approach (surveillance or surgery; if surgery is indicated, limited central or more extended lateral neck surgery).

MTC with central LNM was rare in Group 1. The minimal oncologic risk of microMTC must be balanced with the low risk of central LNM and overtreatment (unnecessary thyroidectomy in CCH with possible complications of bilateral CND). Close surveillance could be an option without missing the opportunity of curing the patient if bCt levels rise and the patient shifts biochemically into the low oncologic risk category (Group 2). The excellent cure rate in Group 2 (definitive diagnosis of MTC) after thyroidectomy and bilateral CND underlines this treatment option. Therefore, delayed thyroidectomy with initial bilateral CND could be recommended in Group 1. To optimize long-term results in patients with a significantly higher oncologic risk documented by higher pretherapeutic bCt levels and a high probability of lateral LNM (Group 3), it is imperative to plan and discuss bilateral LND, in addition to thyroidectomy and bilateral CND beforehand with the majority of patients.

Within this “Ct screening program”, a biochemical long-term cure was achieved in 78.5% (238/303 patients). Based on pretherapeutic bCT, in at least 53.9% (Groups 1 and 2: 165/306 patients), surgery could have been limited to thyroidectomy and CND with a 100% rate of long-term DSS.

## Figures and Tables

**Table 1:** Baseline data and postoperative pathological details of the study population: comparing sporadic (S) and hereditary index (H) patients in the predefined risk groups.

Risk Group Calcitonin pg/mL	Group 1 f: ≤23; m: ≤43			Group 2 f: 24–84; m: 44–99			Group 3 f: ≥85; m: ≥100		
	**S**	**H**	***p***	**S**	**H**	***p***	**S**	**H**	***p***
**Sex**									
f	30	6	0.096	34	5	1.000	73	16	0.638
m	74	5		10	1		45	7	
**Age (years)**									
	60 (51–67)	57 (38–69)	0.453	63 (54–71)	54 (37–58)	0.086	59 (51–68)	53 (44–68)	0.294
**bCt (pg/mL)**									
	15 (10–20)	18 (6–22)	0.571	51 (37–66)	49 (44–73)	0.652	562 (185–2228)	1008 (355–1670)	0.519
**Tumor Ø (mm)**									
	2 (1–4)	2 (1–4)	0.688	5 (4–8)	5 (2–5)	0.186	17 (11–30)	18 (10–25)	0.810
**pT**									
1a	103	11	1.000	41	6	1.000	29	6	0.413
1b	1	0		3	0		40	7	
2	0	0		0	0		33	10	
3a/b	0	0		0	0		11	0	
4a	0	0		0	0		5	0	
**Multiple**									
No	73	3	0.024	42	0	<0.001	108	3	<0.001
Yes	31	8		2	6		10	20	
Unilateral	5	2		0	0		3	0	
Bilateral	26	6		2	6		7	20	
**pN**									
0	102	10	0.262	42	5	0.324	60	9	0.365
1	2	1		2	1		58	14	
1a	2	1		2	1		7	1	
1b	0	0		0	0		51	13	
Unilateral	0	0		0	0		40	9	
Bilateral	0	0		0	0		11	4	
**M**									
0	104	11	-	44	6	-	101	21	0.739
1	0	0		0	0		17	2	
**Stage**									
I	102	10	0.262	42	5	0.324	38	6	0.618
II							22	3	
III	2	1		2	1		6	1	
IVA							35	11	
IVB									
IVC							17	2	

f: female; m: male; S: sporadic; H: hereditary (index); bCt: basal calcitonin; Ø: diameter. T: tumor pathological classification; 1a: T size ≤ 1 cm and intrathyroidal; 1b: T size > 1 cm ≤ 2 cm and intrathyroidal; 2: T size > 2 cm ≤ 4 cm and intrathyroidal; 3a: T size > 4 cm and intrathyroidal; 3b: T gross extrathyroidal extension (sternohyoid, sternothyroid, thyrohyoid, omohyoid muscles); 4a: T gross extrathyroidal extension (subcutaneous soft tissue, larynx, trachea, esophagus, recurrent laryngeal nerve); 4b: T gross extrathyroidal extension (prevertebral fascia) OR encasing the carotid artery, mediastinal vessels. N: lymph nodes pathologically verified; N0: no lymph node metastasis; N1a: lymph node metastasis in the central neck (C; compartment C1a and/or b-Dralle); N1b: lymph node metastasis in the lateral neck. M0: no distant metastasis; M1: distant metastasis radiologically verified. Stage I: T1a, bN0M0; Stage II: T2-3, N0M0; Stage III: T1-3, N1aM0; Stage IVA: T1-3, Nany1bM0; T4aNanyM0; Stage IVB: T4b, NanyM0; Stage IVC: Tany, NanyM1.

## Data Availability

Data are unavailable due to ethical restrictions.

## Supplementary Materials

The following supporting information can be downloaded at https://www.mdpi.com/article/10.3390/cancers16193343/s1, Supplemental Table S1: Risk groups and genotype correlation in the 40 index patients with hereditary MTC; Supplemental Table S2: Clinical and morphological details and follow-up of patients in Group 1 and Group 2 with pN1a or pN0 and persisting/recurrent disease; Supplemental Table S3: Pretherapeutic basal calcitonin level, pTNM classification and frequency and pattern of lymph node metastases [44,45,46,47].
