# Supplementary material for: The Value of Pretherapeutic Basal Calcitonin Cut-Offs for the Therapeutic Strategy and Prediction of Long-Term Outcome of Patients with Medullary Thyroid Cancer—A 30-Year Single-Center Experience
Source: Cancers (Basel). 2024 Sep 29;16(19):3343. doi: 10.3390/cancers16193343 (PMC11482533; doi:10.3390/cancers16193343)
Supplement: Supplementary file 1 [file cancers-16-03343-s001.zip › cancers-3198597-supplementary.pdf]

Supplemental Table S1:

Risk groups and genotype correlation in the 40 index patients with hereditary MTC

Risk group<br>Gender<br>Calcitonin pg/mL		Group 1<br>f: ≤23; m: ≤43	Group 2<br>f: 24-84; m: 44-99	Group 3<br>f: ≥85; m: ≥100	Σ
Exon	Codon	n	n	n	n
8	533			1	1
10	611	2		4	6
	618		1	1	2
	620			1	1
11	634	1	1	5	7
13	768	1	1		2
	790		1	1	2
	791*	3			3*
14	804	1	2	6	9
15	891	2		3	5
16	918**	1		1	2**
		11	6	23	40

MTC: medullary thyroid cancer; f: female, m: male;

*No longer acknowledged as pathogenic mutation [44, 45]

** Exon 16 amino acid exchange: ATG>GTG: Met>Val (!) - causes familial MTC and is suggested clinically a mutation with “moderate risk” [46, 47]

Supplemental Table S2:

Clinical and morphological details and follow-up of patients in Group 1 and Group 2 with pN1a or pN0 and persisting/recurrent disease

	PIN	Gender	Age	bCt (pg/mL)	T		N			M	PTC	FU	Last postCt
					T	R/L mm	∑	N1a	N1b		pTN	(mo)	(pg/mL)
1													
S/1	352	m	61	12	1am	3/2	1/5	1/5	-	0	0	30	<2
S/1	471	m	44	19	1am	8/0	1/166	1/29	R0/91 L0/46	0	1amN0	177	15(R)
H/1	338	f	68	23	1a	6/0	1/3	1/3	-	0	0	150	<2
2													
S/2	592	m	71	55	1a	0/7	1/98	1/28	0/70	0	0	102	<2
S/2	533	f	83	40	1a	6/0		1/12	-	0	1bN0	18	<2
H/2	312	f	57	44	1am	5/3	1/94	1/12	0/72	0	0	146	<2
H/2	327	f	73	73	1am	4/4	0/186	0/16	R0/61 L0/109	0	0	76	8 (P, +)

1: Group 1; 2: Group 2; S: sporadic patient H: hereditary patient; PIN: Patients identity number; m: male; f: female; Age: age at diagnosis in years;
R: right; L: left; B: bilateral; TN: thyroid nodule(s)
T: pathological tumor classification; N: lymph nodes, N1a: central lymph nodes; N1b: lateral lymph nodes; n/n: positive/extirpated nodes
M: distant metastasis
CCH: C-cell hyperplasia; neo: neoplastic; diff: diffuse; nod: nodular; PTC: papillary thyroid carcinoma; FU: follow-up in months (mo);
R: recurrence; P: persisting; +: died (tumor-unrelated)

Supplemental Table S3:

Pretherapeutic basal calcitonin level – pTNM classification – frequency and pattern of lymph node metastases

**Risk Group** Gender bCt pg/mL		**Group 1** f: ≤23; m: ≤43					**Group 2** f:24-84; m: 44-99					**Group 3** f: ≥85; m: ≥100					∑	%
		**Minimal oncologic risk**					**Low oncologic risk**					**High oncologic risk**						
	n	115 (37.6)					50 (16.4)					141 (46.1)					**306**	100
**T**		**1a**	**1b**	**2**	**3a/b**	**4a**	**1a**	**1b**	**2**	**3a/b**	**4a**	**1a**	**1b**	**2**	**3a/b**	**4a**		
	n/n %	114/115 99.1	1/115 0.9	0	0	0	47/50 94	3/50 6	0	0	0	35/141 24.8	47/141 33.3	43/141 30.5	11/141 7.8	5/141 3.5	**306**	**100**
**N**	0	111	1	-	--	-	44	3	-	-	-	23	21	23	2	0	228	74.5
		112/115 (97.4)					47/50 (94)					69/141 (48.9)						
	1	3/114	0/1				3/50	0	-	-	-	12/35 34.3	26/47 55.3	20/43 46.5	9/11 81.1	5/5 100	**78**	**25.4**
		3/115 (2.6)					3/50 (6)					72/141 (51.1)						
	1a	3	0	-	-	-	3	0	-	-	-	0	3	5*~	0	0	6+8	5.7
	1b	0	0	-	-	-	-	-	-	-	-	12	23	15	9	5	64	45.4
	L only											5*	7	2	1	0	15	
	C+UL											5**	13*	9***	4*	3***	34	
	C+BL											2**	3	4*	4***	2*	15	
**M**	1											5++	1	5+	4+++	4+	**19**	**6.2**

f: female; m: male; bCt: basal calcitonin;
T: Tumor – pathological classification; 1a: T size ≤ 1 cm and intrathyroidal; 1b: T size > 1 cm ≤ 2 cm and intrathyroidal; 2: T size > 2 cm ≤ 4 cm and intrathyroidal; 3a: T size > 4 cm and intrathyroidal; 3b: T gross extrathyroidal extension (sternohyoid, sternothyroid, thyrohyoid, omohyoid muscles); 4a: T gross extrathyroidal extension (subcutaneous soft tissue, larynx, trachea, esophagus, recurrent laryngeal nerve); 4b T gross extrathyroidal extension (prevertebral fascia) OR encasing the carotid artery, mediastinal vessels
N: Lymph nodes (LN)– pathologically verified; N0: No lymph node metastasis (LNM); N1a: lymph node metastasis in the central neck (C; compartment C1a and/or b - Dralle); N1b: lymph node metastasis in the lateral neck; L only: skip LNM; C+UL: central and unilateral LNM; C+BL: central and bilateral LNM
M0: No distant metastasis; M1: Distant metastasis – radiologically verified; *: Patient with M1: n=19; **: 2 patients with M1; ***: 3 patients with M1; ~M1 – no LND performed!! +: Patient with C+BL: n=7; ++: 2 patients with C+BL; +++: 3 patients with C+BL.
